# Comparing the effects of intravenous and subcutaneous Erythropoietin on blood indices in hemodialysis patients

**DOI:** 10.1038/s41598-018-38193-z

**Published:** 2019-02-19

**Authors:** Faramarz Ghasemi, Alireza Abdi, Nader Salari, Mohammad Reza Tohidi, Aazam Faraji

**Affiliations:** 1 Faculty of Nursing and Midwifery, student’s research committee, Kermanshah University of Medical Sciences, Kermanshah, Iran; 2 School of Nursing and Midwifery, Kermanshah University of Medical Sciences, Kermanshah, Iran; 3 Urology and Nephrology Research Center, Kermanshah University of Medical Sciences, Kermanshah, Iran

## Abstract

Erythropoietin is used routinely in hemodialysis patients to increase hemoglobin levels in patients with ESRD and anemia. Considering the importance of anemia in hemodialysis patients and its complications, in this study, the effect of erythropoietin administration on blood parameters by comparing the methods of subcutaneous and intravenous administrations was investigated. The research was done as a retrospective descriptive-analytic study. The subjects were 300 hemodialysis patients receiving erythropoietin in two groups (intravenous and subcutaneous) from hospitals affiliated to Kurdistan and Kermanshah University of Medical Sciences. Data were extracted from the patients’ records and entered into a researcher-made checklist during 6 months and analyzed using SPSS version 20 and descriptive and inferential statistics. The results showed that there was a significant difference between the mean rank of hematocrit in subcutaneous and intravenous injections (P-value = 0.002) and it was higher in the subcutaneous injection group. Also, independent t-test showed that there was a significant difference between the mean of hemoglobin concentration among subcutaneous and intravenous injections (P-value = 0.001) and it was higher in the subcutaneous injection group. However, there was no significant difference between the mean of ferritin in both groups (P-value = 0.084). According to the results, the injection of erythropoietin in the subcutaneous method was more effective than intravenous. It is recommended to use this approach to improve blood indices in hemodialysis patients.

## Introduction

Chronic kidney disease (CKD) covers a range of different pathological processes associated with abnormal function of the kidney and gradual decrease of glomeruli filtration that leads to end stage renal disease (ESRD)^1^. The term ERSD is used when about 90% of kidney function is lost and the body fails to keep the water and electrolyte balance, dispose of waste materials, and keep the normal function of hormones^2^. There are different methods to treat kidney failure patients and hemodialysis is one and the most common of them^3^.

Hemodialysis patients suffer a wide range of problems and side-effects and anemia in these patients are frequently mentioned in lab reports^3^. Although, other patients with renal function failure demonstrate the symptoms of anemia, the severity of the disorder in advanced renal failure patients on chronic hemodialysis is higher^4^. Azhir *et al*.^5^ reported anemia in 82% of hemodialysis patients and 48% of the cases was severe anemia^5^. Anemia, according to the World Health Organization (WHO), is defined as a hemoglobin level less than 13 g/dl in men and postmenopausal women, and 12 g/dl in women before menopause. The majority of patients (90%) with glomerular filtration rate (GFR) of 25 ml/min are diagnosed as an anemia case since their hemoglobin level is less than 10 g/dl. Initiation of anemia in chronic renal failure patients is relatively faster, so that it starts when GFR is at 25–30 ml/min range^6^.

Anemia in renal failure patients can gradually lead to several physiological disorders such as hypoxia, cardiomegaly, increased cardiac output, hypertrophy of ventricles, angina, heart failure, cognitive and cerebral disorders, immune system failure and changes in menstrual cycle^7,8^. Anemia in children might lead to growth disorders and attenuation of creativity and intelligence. In general, anemia degrades quality of life and decreases life expectancy^9^.

Nowadays, erythropoietin is one of the main bases of anemia treatment in patients with renal failure^10^. It is a glycoprotein hormone that is secreted in adults, mostly by the kidney and by the liver to a less extent. The hormone stimulates the bone marrow to produce red blood cells. The body’s response to erythropoietin depends on dosage, type of administration, and frequency of administration^11^.

There are ongoing debates about the relationship between the effectiveness of intravenous (IV) and subcutaneous (SC) erythropoietin administrations. Researchers believe that a lower dosage of the drug is needed in SC administration while its effects last for a longer period of time. On the other hand a higher dosage of the drug is needed in the IV method^10^. There are inconsistent clinical and research evidences in this regard. For instance, Trakarnvanich *et al*.^12^ reported that the difference between hematocrit level with the IV and SC administrations was not significant^12^. Daniel *et al*. (2015) on the other hand reported that comparing with IV administration, SC administration in the patient on hemodialysis resulted in better outcomes in terms of mortality rate, cardiovascular side-effects, and hospitalization period^10^. In addition, Farlan *et al*.^13^ showed that changing the administration method from SC to IV method needed higher dosage of erythropoietin and led to higher blood iron and transfusion^13^. Some assumptions have raised in Table 1.

**Table 1:** Assumptions about erythropoietin injection.

1- There are a controversies about injection route of erythropoietin in hemodialysis patients
2- SC injection of erythropoietin is accompany with lower side effects
3- The required dose of erythropoietin to take clinical outcome is more in IV injection in hemodialysis patients

Knowing that the nurses’ knowledge as to storing, preparing, and administering erythropoietin can be effective on efficiency of the drug in hemodialysis patients^14^ and that the debate on the optimum way of administering the drug is not concluded, the present study is aimed for comparing the effects of IV and SC erythropoietin on the blood indices of hemodialysis patients. The research hypothesis was: 1- there are a different between hemodialysis patients with SC and IV injection of erythropoietin in term of hemoglobin and hematocrit and ferritin.

## Methods

This analytical-descriptive cohort retrospective study was carried out from December 2017 to May 2018 in the hospitals associated with Kurdistan and Kermanshah universities of medical sciences and the study was approved by the research deputy of Kermanshah University of medical sciences and its research ethics with ID No.: IR.KUMS.REC.1397.063, also All the methods were performed in accordance with the determined guidelines and regulations. The sampling was done through census method. So that due to a limitation in the number of samples, all the patients who met the inclusion criteria (n = 300) were examined. Inclusion criteria were age above 18 years, no history of hepatitis, malignancy, and epilepsy, adequate iron level (ferritin >100 ng and transferrin saturation >20%), and using one administration method over the past six months (IV or SC). Exclusion criteria were hyperparathyroidism (Parathyroid hormone or PTH > 600), and incomplete medical file.

Data gathering tools included a demographic information form and a checklist. The demographic information form included items of the age, gender, marital status, education level, and job. The checklist covered therapeutic and treatment process information by questions about hematocrit level, hemoglobin report at different occasions (during last 6 months), hemodialysis record (during last 6 months), and the number of hemodialysis per week. To design of the checklist, nursing literature and published papers were used. For content validity, the checklist was provided to 10 faculty board members and experts and their comments were implemented in the checklist.

After approval of the research project and making the arrangements like securing a license from the Research deputy of Kermanshah University of Medical Science, and also securing a permit for officials of the hospitals, the authors attended the hospitals in Kermanshah (Imam Reza and Imam Khomeini hospitals) and Kurdistan (Touhid - Sanandaj, Sina - Kamyaran, and Bu Ali – Marivan, Imam Khomeini – Saghez, Salahedin Ayoubi – Bane). The participants, who had inclusion criteria, were recruited to the study and the objectives and design of the study were explained to them and an informed written consent was signed before data gathering. Therefore, after making arrangements with discharge department, documents department, and dialysis ward of the hospitals, the researchers selected a number of hemodialysis patients based on the inclusion criteria and through convenience sampling method. Afterwards, the subjects were categorized into two groups (IV and SC groups). The drug used in the study was supplied by the same company. Afterward, medical information about the subjects was collected using the checklist. The data was analyzed by SPSS-20 software using descriptive (mean, mean rank, frequency, frequency percent), and inferential statistics such as independent t-test (for determining the difference between mean of two groups in parametric quantitative variables), Mann Whitney U (for assessing the difference between two groups in term of non-parametric quantitative variables), and repeated measures Analysis of variance or ANOVA (for comparing the mean of Hemoglobin or Hematocrit during 6 months in two groups), while the sphericity was determined by Mauchly’s test which was significant (P < 0.001) for both variables, so Greenhouse Geisser was reported, because it was less than 0.75^15^. Bonferroni test was applied to the post hoc test. We also used two-way repeated measure ANOVA for clarify if the changes are related to between subjects effects of duration (6 months) and type of injection (IV and SC). Shapiro-wilk test was applied for determining the normality of the quantitative variables, and the homogeneity of the variance in independent t-test was checked by Levene’s test. The significant level of the tests was considered less than 0.05.

### Ethics approval and consent to participate

The study was approved by research ethics committee of Kermanshah University of medical sciences, Kermanshah-Iran under ID No.: IR.KUMS.REC.1397.063.

## Findings

As the results showed, 59.3% of the patients were men, 37.3% were unemployed, 57% were illiterate, and 73% lived in a city. In 53.7% of the cases the access vein was through the fistula venous. Moreover, 28% suffered diabetes and 55.7% were on dialysis due to hypertension. Eighty-six percent of the patients had three hemodialysis sessions per week. Additionally, the mean and standard deviation (SD) of age of the patients was 57.22 ± 14.70 years and mean and SD of duration of hemodialysis was 43.32 ± 32.08 months. There was no any difference between two groups in term of demographic characteristics (Table 2). The Shapiro-wilk test showed that hemoglobin and hematocrit during six months had a normal distribution (P > 0.05), but ferritin level and mean of hematocrit (the summation of 6 months), age and hemodialysis duration were non-normal (P-value < 0.05). The Levene’s test showed a good homogeneity for hemoglobin and hematocrit variables in all measures.

**Table 2:** Relative and absolute frequency distribution of demographics variables of the subjects of two groups.

Variables	Total Frequency (%)	IV group Frequency (%)	SC group Frequency (%)	Statistical test
**Gender**				*K2 = 2.026
Male	178 (59.3)	116 (65.2)	62 (34.8)	P-value = 0.155
Female	233 (40.7)	89 (73)	33 (27)	
**Education**				*K2 = 4.579
Illiterate	171 (57)	125 (73.1)	26 (26.9)	P-value = 0.101
Junior high school	96 (32)	61 (63.5)	35 (36.5)	
Diploma	33 (11)	19 (57.6)	14 (42.4)	
**Occupation**				*K2 = 6.083
Unemployed	101 (33.7)	64 (63.4)	37 (36.6)	P-value = 0.108
Housewife	102 (34)	79 (77.5)	23 (22.5)	
Office employee	42 (14)	26 (61.9)	16 (38.1)	
Freelancer	55 (18.3)	36 (65.5)	19 (34.5)	
**Domicile**				*K2 = 0.549
City	219 (73)	147 (67.1)	72 (32.9)	P-value = 0.459
Village	81 (27)	58 (71.6)	23 (28.4)	
**Access to vein**				*K2 = 2.305
Fistula	161 (53.7)	106 (65.8)	55 (34.2)	P-value = 0.316
Graft	6 (2)	3 (50)	3 (50)	
Catheter	133 (44.3)	96 (72.2)	37 (27.8)	
**Cause of hemodialysis**				*K2 = 5.363
Hypertension	167 (55.7)	123 (73.7)	44 (26.3)	P-value = 0.068
Diabetes	75 (25)	48 (64)	27 (36)	
Other	58 (19.3)	34 (58.6)	24 (41.4)	
**Hemodialysis sessions per week**				*K2 = 0.334
≤2	42 (14)	26 (61.9)	16 (38.1)	P-value = 0.933
3	258 (86)	179 (69.4)	7 (30.6)	
**Age (years)** **mean ± SD**	57.22 ± 14.70	57.25 ± 14.61	57.15 ± 14.96	**Z = 0.283 P-value = 0.777
**Dialysis period (month)** **Mean ± SD**	43.32 ± 32.08	44.80 ± 31.33	49.58 ± 33.58	**Z = 1.321 P-value = 0.187

*K2 = chi-squared.

**Z = mann whitney U test.

**Figure 1:**
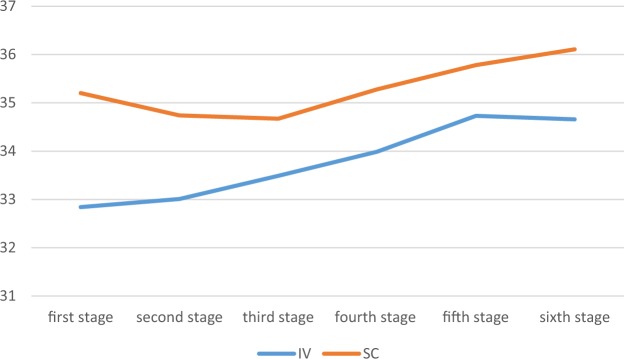
Hematocrit change trend from stage one to stage six based on the type of injection.

Independent t- test showed a significant difference between IV and SC groups in hematocrit during six months (Table 3), and also the mean of them was higher in the SC group (Z = −3.06, P-value = 0.002) (Table 4). The changes are mentioned in Fig. 1.

**Table 3:** Mean level of hematocrit in the subjects at first-six stages of injection based on IV and subcutaneous administration.

Hematocrit mean/stages	IV	SC	Statistics
	Mean ± SD	Mean ± SD	
Stage 1	32.84 ± 4.94	35.20 ± 4.41	*t = −3.965 p-value = 0.001
Stage 2	33.01 ± 4.60	34.74 ± 4.02	*t = −3.150 p-value = 0.002
Stage 3	33.49 ± 4.61	34.67 ± 4.92	*t = −2.020 P-value = 0.002
Stage 4	33.99 ± 4.77	35.28 ± 4.44	*t = −2.219 p-value = 0.044
Stage 5	34.73 ± 5.21	35.78 ± 4.76	*t = −1.671 p-value = 0.096
Stage 6	34.66 ± 4.83	36.11 ± 4.53	*t = −2.547 p-value = 0.015
Statistics	**F = 42.03 p-value = 0.001	**F = 8.14 P-value = 0.149	

*t = independent t-test.

**F = repeated measures ANOVA.

**Table 4:** Means level of hematocrit based on injection technique.

IV		SC		Statistics
Mean ± SD	Mean rank	Mean ± SD	Mean rank	
33.79 ± 3.76	140.06	35.30 ± 3.27	173.04	*Z = −3.063 p-value = 0.002

*Z = mann whitney U test.

**Figure 2:**
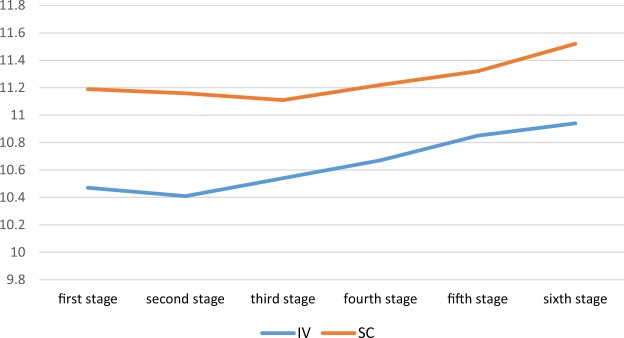
Hemoglobin change trend from stage one to stage six based on the type of injection.

In addition, independent t-test showed that there was a significant difference between the IV and SC groups during 6 months (P-value < 0.05) and the mean of total hemoglobin level (P-value = 0.001), so that the SC group had higher hemoglobin (Table 5). Figure 2 shows Hemoglobin change trend from stage one to stage six based on the type of injection

**Table 5:** Mean level of hemoglobin in hemodialysis patients from stage one to stage six based on the type of injection.

Hemoglobin mean level	IV	SC	Statistics
	Mean ± SD	Mean ± SD	
Stage 1	10.47 ± 1.72	11.19 ± 1.54	t = −3.455 p-value = 0.001
Stage 2	10.41 ± 1.61	11.16 ± 1.38	t = −3.921 p-value = 0.001
Stage 3	10.54 ± 1.58	11.11 ± 1.52	t = −2.941 p-value = 0.004
Stage 4	10.67 ± 1.62	11.22 ± 1.42	t = −2.838 p-value = 0.005
Stage 5	10.85 ± 1.64	11.32 ± 1.53	t = −2.337 p-value = 0.020
Stage 6	10.94 ± 1.63	11.52 ± 1.42	t = −2.955 p-value = 0.003
Statistics	F = 7.97 P-value < 0.001	F = 1.71 P-value = 0.155	
**Hemoglobin (mean of 6 stages)**	**IV** **Mean** ± **SD**	**SC** **Mean** ± **SD**	**Statistics**
	10.6 ± 1.30	11.25 ± 1.08	t = −3.940 p-value = 0.001

**Figure 3:**
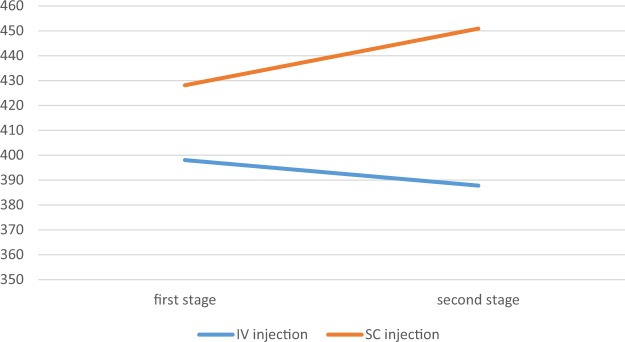
Ferritin change trend from stage one to stage two based on the type of injection.

The Mann Whitney U test also indicated no significant difference between the two groups in terms of ferritin level (P-value = 0.056) (Table 6). Figure 3 represents ferritin change trend between stage one and two based on the type of injection.

**Table 6:** Mean level of ferritin in hemodialysis patients from stage one to stage two based on the type of injection.

Ferritin stage	IV		SC		Statistics
	Mean ± SD	Mean rank	Mean ± SD	Mean rank	
Stage 1	398.05 ± 216.49	146.14	428.11 ± 206.87	159.92	Z = −1.280 p-value = 0.201
Stage 2	387.75 ± 209.83	140.20	450.89 ± 208.79	168.42	Z = −2.436 P-value = 0.015
Statistics	Z = −0.078 P-value = 0.938		Z = −2.650 P-value = 0.008		
**Ferritin (total)**	**Mean** ± **SD**	**Mean rank**	**Mean** ± **SD**	**Mean rank**	**Statistics**
	392.90 ± 197.36	143.17	439.50 ± 195.27	164.17	Z = −1.914 p-value = 0.056

The repeated measure ANOVA showed a significant different during 6 months regarding the variables of hematocrit for IV (F = 12.19, P-value = 0.001), and SC (F = 2.60, P-value = 0.04) (Table 2), two-way repeated measure ANOVA, which assessed the between subject effect of type of injection and duration, was significant (F = 11.29, P-value = 0.001), showing dependency of the changes to the type of injection. The changes of hematocrit during 6 months have been represented in Fig. 1. Table 7 shows the Benneferoni results of the mean differences of hematocrit between measures.

**Table 7:** comparing the mean difference between 6 measures of hematocrit in IV and SC groups.

Types of injection	SC		IV	
month	Mean difference	P value	Mean difference	P- value
1 and 2	0.460	0.197	−0.162	0.481
1 and 3	0.529	0.347	−0.163	0.045*
1 and 4	−0.080	0.867	−1.148	0.001*
1 and 5	−0.584	0.313	−1.815	<0.001*
1 and 6	−0.908	0.132	−1.818	<0.001*
2 and 3	0.069	0.868	−0.481	0.055
2 and 4	−0.540	0.166	−0.986	0.005*
2 and 5	−0.044	0.050*	−1.723	<0.001*
2 and 6	−1.368	0.014*	−1.656	<0.001*
3 and 4	−0.609	0.169	−0.504	0.088
3 and 5	−1.114	0.044*	−1.242	0.001*
3 and 6	−1.438	0.017*	−1.174	0.001*
4 and 5	−0.504	0.223	−0.738	0.014*
4 and 6	−0.828	0.104	−0.670	0.029*
5 and 6	0.324	0.458	0.068	0.796

*Is significant.

For hemoglobin, there was a change in the IV group (F = 7.97, P-value < 0.001) during 6 months, but it was not significant about SC injection (F = 1.71, P-value = 0.155) (Table 5). The Bonferroni test for IV group is presented in Table 8.

**Table 8:** comparing the mean difference between 6 measures of hemoglobin in IV group.

Types of injection	IV	
month	Mean difference	P-value
1 and 2	0.063	0.410
1 and 3	−0.069	0.520
1 and 4	−0.199	0.93
1 and 5	−0.382	0.002*
1 and 6	−0.470	<0.001*
2 and 3	−0.133	0.131
2 and 4	−0.262	0.028*
2 and 5	−0.446	<0.001*
2 and 6	−0.534	<0.001*
3 and 4	−0.129	0.216
3 and 5	−0.313	0.009*
3 and 6	−0.401	0.001*
4 and 5	−0.184	0.036*
4 and 6	−0.272	0.006*
5 and 6	−0.088	0.251

*Is significant.

## Discussion

In this study, the effects of IV and SC injections of erythropoietin on blood indices of hemodialysis patients were compared. As the results indicated, SC method was more effective in blood indices so that the differences between the two groups were significant in terms of the hematocrit and hemoglobin levels. Comparison of the mean scores between the two groups indicated that hematocrit in the SC group was higher and the hemoglobin level was higher in the SC group. However, although the ferritin level was higher in the SC group, Mann Whitney test indicated not significant difference between the two groups. Thereby SC erythropoietin was more effective than IV erythropoietin in the hemodialysis patients.

Daniel *et al*. (2015) carried out a study titled “the relationship between erythropoietin dosage and administration technique in hemodialysis patients in the USA” and studied 62710 patients on hemodialysis in USA-based clinics between 1997 and 2005. The study was carried out as a cohort retrospective study. They found that IV erythropoietin dosage to achieve the same effects as with one unit of SC erythropoietin must be 25% higher. The authors reported that the increase in hemoglobin in SC administration was higher than the IV method^10^. Despite the differences in methodology, their results are consistent with the present study.

Rahimian *et al*.^16^ studied 60 patients with renal failure selected through convenience sampling method in the dialysis ward of Shahid-Rahnemoun Hospital Yazd with two months follow up. They measured hemoglobin level of the patients one week before the study, one week and one month after initiation of the intervention, and two months after the study. The mean increase in hemoglobin was 1.48 g/dl (P-value < 0.05)^16^. The only index under study was hemoglobin level. They also examined the effects of erythropoietin regardless of the way of administering the drug.

In the retrospective study on comparing IV and SC administration of a recombinant form of erythropoietin, Trakarnavich *et al*. (2004) studied medical file of 60 patients in some of Bangkok-based clinics. The subjects were patients whose hematocrit level had been fixed over a three month period using SC erythropoietin and then shifted to IV erythropoietin for another six months. They reported that the mean hematocrit level using SC erythropoietin was 30.49 ± 4.21 for at least three months and it was 30.24 ± 4.99 during the next three months using IV erythropoietin, and the differences were not significant^12^. Their study is different from the present study in terms of methodology. Here two groups of patients (IV and SC erythropoietin) were under study while they focused on only one group of patients whose erythropoietin administration method had been changed. In addition, the type erythropoietin used in these two studies was not the same. These differences may explain the inconsistent results.

There are studies on the role of ferritin in response of the body to erythropoietin in hemodialysis patients. Failure to take the role of resistance causing factors and serum ferritin in particular, notably attenuates the effect of erythropoietin. Kausz *et al*. reported that what follows the bone marrow stimulation using erythropoietin is a secondary iron deficiency that is caused by rapid consumption of iron resources of the body. Ensuring iron balance in the patients is the main factor in obtaining desirable results by erythropoietin and achieving the target hematocrit level^17^.

A descriptive study by Shariati *et al*.^18^ examined the prevalence of iron deficiency anemia in hemodialysis patients. They reported that the frequency of anemia in the hemodialysis patients was 57.6%, iron deficiency anemia was observed in 31.1%, and 29.16% of the subjects had transferrin saturation of less than 20%. No significant relationship was found between the symptom of anemia, time period of dialysis, the causes of disease, and iron deficiency anemia (p = 0.06). Functional iron deficiency was significantly related to receiving erythropoietin (P > 0.001)^18^. According to the results of this study, changes in the mean level of ferritin were significant after six months in the SC groups; although, the difference between the two groups was not significant. The results here support the hypotheses and SC erythropoietin is more effective than IV erythropoietin.

## Conclusion

In this study SC method of erythropoietin administration was more effective on hematologic indexes such as hematocrit and hemoglobin, herewith, because the nurses’ knowledge about storing, preparing, and administering erythropoietin is effective in the efficiency of the drug, having a deeper insight into the most effective method, SC administration is an effective step toward improvement of the blood indices in hemodialysis patients. As to the limitations of the study, incomplete medical files and the small number of the samples are notable. Further studies with larger sample groups and different doses of erythropoietin to achieve results with more reliability are recommended.

## Data Availability

The datasets analyzed during the current study are available from the corresponding author on reasonable request.
